# Supporting local planning and budgeting for maternal, neonatal and child health in the Philippines

**DOI:** 10.1186/1478-4505-11-3

**Published:** 2013-01-23

**Authors:** Sophie La Vincente, Bernardino Aldaba, Sonja Firth, Aleli Kraft, Eliana Jimenez-Soto, Andrew Clark

**Affiliations:** 1 Centre for International Child Health, Murdoch Childrens Research Institute, University of Melbourne, University of Melbourne Education Offices, East Level 2, Royal Children’s Hospital, Flemington Road, Parkville, VIC 3052, Australia; 2 UPecon Foundation, Inc. Room 322, UP School of Economics, University of the Philippines, Diliman, Quezon City, 1101, Philippines; 3 School of Population Health, University of Queensland, 4th Floor, Public Health Building, Herston Road, Herston, QLD, 4006, Australia; 4 Department of Health Services Research and Policy, London School of Hygiene and Tropical Medicine, 15-17 Tavistock Place, London, WC1H 9SH, UK

**Keywords:** Evidence-based planning and budgeting, Maternal, Neonatal and child health, The Philippines, Sub-national health planning, Health system constraints

## Abstract

**Background:**

Responsibility for planning and delivery of health services in the Philippines is devolved to the local government level. Given the recognised need to strengthen capacity for local planning and budgeting, we implemented Investment Cases (IC) for Maternal, Neonatal and Child Health (MNCH) in three selected sub-national units: two poor, rural provinces and one highly-urbanised city. The IC combines structured problem-solving by local policymakers and planners to identify key health system constraints and strategies to scale-up critical MNCH interventions with a decision-support model to estimate the cost and impact of different scaling-up scenarios.

**Methods:**

We outline how the initiative was implemented, the aspects that worked well, and the key limitations identified in the sub-national application of this approach.

**Results:**

Local officials found the structured analysis of health system constraints helpful to identify problems and select locally appropriate strategies. In particular the process was an improvement on standard approaches that focused only on supply-side issues. However, the lack of data available at the local level is a major impediment to planning. While the majority of the strategies recommended by the IC were incorporated into the 2011 plans and budgets in the three study sites, one key strategy in the participating city was subsequently reversed in 2012. Higher level systemic issues are likely to have influenced use of evidence in plans and budgets and implementation of strategies.

**Conclusions:**

Efforts should be made to improve locally-representative data through routine information systems for planning and monitoring purposes. Even with sound plans and budgets, evidence is only one factor influencing investments in health. Political considerations at a local level and issues related to decentralisation, influence prioritisation and implementation of plans. In addition to the strengthening of capacity at local level, a parallel process at a higher level of government to relieve fund channelling and coordination issues is critical for any evidence-based planning approach to have a significant impact on health service delivery.

## Background

With an estimated population of over 92 million people, the Philippines is one of the most populous countries in the world. Overall, child health has improved in recent years; however, the rate of decline of infant and under-five mortality has slowed, and maternal and neonatal mortality has declined little since the 1990s [1,2]. Under the decentralised system of the Philippines, the delivery of health services has been devolved to local governments (municipalities, provinces and cities) [3]. These local government units (LGUs) are now responsible for developing their own health plans and budgets, and securing the corresponding financial resources. However, limitations in their capacity to undertake these tasks have been recognised as one of the impediments to implementing national reforms such as universal health coverage [4].

In response to the problem of local level capacity, the National Department of Health (DOH) introduced the concept of Province-wide Investment Plans for Health (PIPH), requiring local plans to be proposed to a central appraisal committee. Insufficient central level capacity to provide direct technical support to the 82 provinces and limited roles for regional health offices in the early years hampered the original aim of this process. In more recent years, delegating technical assistance and financial responsibilities to regional health centres attempted to address these short-comings; however, a mechanism to improve capacity for strategic problem-solving and a formal approach to support evidence-based health planning and budgeting at local level was also required.

Here we describe the experience of implementing an initiative to support local planning, prioritisation and budgeting for maternal, neonatal and child health (MNCH) in a number of sub-national units in the Philippines. In partnership with local health officials, we implemented the Investment Case (IC) approach in two mainly rural provinces (Northern Samar, population 670,000; and Eastern Samar, population 440,000), and one highly-urbanised city (Pasay City, population 410,000). This approach engages local policymakers and planners in structured problem solving to identify key health system constraints to the provision and uptake of MNCH interventions, and develop strategies to overcome identified problems. A decision-support model that estimates the cost and impact of alternative approaches is used to guide the selection and prioritisation of strategies [5]. We outline how the initiative was implemented, the aspects that worked well, and the key limitations identified in the sub-national application of this approach.

## Methods

The cornerstone of the IC approach is strategic problem-solving for MNCH planning and budgeting [5]. The process starts with a critical assessment of the available evidence on six parameters related to the performance of health systems; three on the supply side, two on the demand side, and one on quality [6]. Indicators for each of these parameters are site-specific and are defined for a range of critical MNCH services by the local health officials, in consultation with the IC research team and guided by local data availability. Using the evidence collated, local health planners, providers and other stakeholders are engaged in a structured, systematic examination of the key constraints hampering the scaling-up of priority MNCH interventions in disadvantaged populations. After key stakeholders have identified the root cause of the problems, feasible strategies required to address those constraints are identified, and the resultant increases in coverage of relevant health services are estimated. An epidemiological and economic decision-support model that builds on the conceptual framework of bottleneck analysis [5] is used to estimate the impact and the associated costs of the different strategies. The model includes sixty six interventions for which there is global evidence on efficacy to reduce the burden of MNCH mortality [7-9]. These interventions cover the continuum of care, from pre-pregnancy interventions such as family planning, to childhood interventions such as use of antibiotics for pneumonia. Finally, the estimated impact and cost of the different identified strategies are then reviewed to guide decision-making for planning and priority setting.

To ensure the IC findings would be able to support the development of local MNCH plans and budgets, it was important to involve government stakeholders at all stages of the process [10]. Meetings were first held with DOH for endorsement and approval of the process and for site selection. The DOH identified two provinces (Northern Samar and Eastern Samar) and one city (Pasay City) in which to implement the IC. The selection was driven by two types of considerations: i) Capacity to benefit: these were considered to be LGUs where assistance with planning would be most beneficial, and where the LGU health official is responsive and motivated; ii) Equity: Northern Samar and Eastern Samar are among the poorest provinces in the Philippines, and experience some of the worst MNCH outcomes. The rapid growth of cities in the Philippines has led to significant urban poverty [11], presenting specific challenges to the delivery and uptake of MNCH services amongst the poor. Overall statistics suggest that Pasay City residents experience better MNCH outcomes relative to national averages; however, as is typically observed in cities, such statistics mask great inequity [2]. Despite the close proximity of health services, for the most disadvantaged residents of Pasay City there remain significant barriers to accessing key MNCH interventions.

Following site identification, meetings were then held with the relevant regional health offices, and with provincial and city health officials of the three proposed LGUs to discuss the approach and gauge local interest in implementing the process. The initiative was considered and accepted as an approach that had potential to provide inputs to each LGU’s five-year Health Investment Plans and/or Annual Operational Plans (AOP).

## Results

### Data collection and validation

Following the decision by local health officials to participate in the initiative, the research team worked together with province and city health office staff to identify, collate and map sources of data that could be used in the analysis [12]. This included data sources and previous analytical work at the local, regional and national level in relation to population demographics, mortality rates and causes, intervention coverage, and local health system structure and costs. We identified and reviewed routine health information systems, special surveys and studies, technical documents, guidelines and previous analytical work. Other sources of data included national agency publications, such as national health insurance reports, health budget/expenditure, policy issuances, guidelines, and protocols. The initial round of data collection took place between February and December 2009; however, data collection and validation was ongoing throughout the implementation process to ensure the best and most appropriate data for the analysis were used. The limited data available at local level was a significant challenge, particularly in relation to mortality rates and causes, health service coverage parameters, and health system costs.

While mortality rates were generally available at the province /city level (with the exception of neonatal mortality, for which data were much scarcer), estimates were typically drawn from vital registration or death review systems that are recognised to suffer from underreporting. Data on causes of death among neonates (from delivery to one month of age) and children aged 1 to 59 months were available at the national level only. We sought expert validation of the available mortality data to verify whether these estimates were considered locally representative.

In reviewing health service coverage for critical MNCH interventions, indicators were defined across parameters of supply, demand/use and quality. Local data were generally available for the supply side parameters, such as the proportion of health facilities with a continuous supply of relevant commodities or in which trained staff were located. Most of these data were drawn from local health information systems, such as the Field Health Service Information System which records provision of health services. However, there were little or no reliable local data available for indicators of demand/access and quality, which require information on population use of health services. For example, health programs record and report on how many children with acute respiratory infection (ARI) are seen and treated with antibiotics, but there is no information available on the total number of children in the population who experience an ARI, which is needed in order to estimate the coverage of antibiotic treatment of childhood ARI. We relied on regional and national data sets to substitute for local data. For example, we used regional estimates from national surveys such as the household-based National Demographic and Health Survey and Family Planning Survey. Generally, these estimates are representative down to the regional level, not to the province/city level. Where possible, triangulation of data from different sources was undertaken to validate inputs for use in the analysis, and we sought expert validation to verify the estimates used.

It was difficult to obtain information on services provided at public hospitals. Management of many hospitals is outside of the Provincial or City Health Office, and public health officials (e.g. Provincial, City and Municipal Health Officers) often do not have ready access to the information systems of such hospitals. In addition, little information could be obtained on the private sector, which plays a particularly significant role in provision of MNCH services in urban areas. For example, four of the five facilities offering basic emergency obstetric care in Pasay City were operated by private providers. This demonstrates the difficulty faced by cities in planning services and assessing the need for quality improvement initiatives when a significant number of providers are private and the local government has no access to data from these facilities and limited regulation over their practice. For a number of indicators we gathered data directly from hospital records.

Information on the cost of health system inputs is critical for the development of sound plans, but many costs were not routinely available to LGUs for their use in planning. We gathered cost information from a wide range of sources, including distributor/suppliers’ price lists and consulting key informants regarding prices of some commodities.

### The problem-solving approach

Problem-solving workshops were facilitated by the IC team and representatives from the DOH regional health offices. Between two and three problem-solving workshops were held in each LGU over a five-month period. Each workshop was one to two days in duration. Participants included program managers from the provincial/city health office, municipal health officers, health centre personnel, district nurse supervisors, sanitary inspectors and other key personnel. At these workshops, local government officials participated in structured discussion to identify the key constraints to scaling-up MNCH intervention coverage and develop a range of strategies to overcome those constraints.

The participation of stakeholders from all levels of the local health system provided rich discussion, drawing on varied experiences and perspectives. For example, municipal health workers provided the perspectives of those operating on the ground and their contributions acted as a “reality check” on the feasibility of proposed strategies or increased coverage targets. The participation of regional staff as facilitators proved valuable in the development of strategies and solutions as they were able to refer to experiences in other provinces in solving problems similar to those being discussed; and to explain the content of recent national policies and protocols. A number of scenarios that included different combinations of the strategies defined during the workshops were modelled using the decision-support tool. This provided an estimate of cost of each scenario and the anticipated impact. These results were then used by local officials to determine which combination of strategies represented the “best buys”, that is, the scenarios likely to deliver the greatest anticipated impact given the resources required.

### Adoption of recommended strategies

The majority of the strategies recommended by the IC were accepted by the three LGUs and the IC team assisted in translating the strategies into the format required for inclusion in local plans and budgets, including identification of different funding sources. The IC team then verified that these strategies had indeed been incorporated into the plans and budgets. A subsequent follow-up review of the initiative validated that the majority of strategies had been included in the 2011 plans of the three LGUs, and changes in their budgets had been made to reflect these strategies [13]. However, this review also revealed that a key strategy developed by Pasay City for which there had been initial enthusiasm and high expectations, was no longer being supported.

## Discussion

The detailed review of available local data undertaken for the IC highlighted the deficits in information that can be used effectively for planning at the sub-national level. Rather than creating a sense of futility in the use of evidence for planning, this recognition focused health officials on understanding how to improve their local health information system to enhance future planning and budgeting, and to enable more effective monitoring. Indeed, recognising the gaps in local data prompted health officials in Pasay City to allocate funding in their plans for the recruitment of a data management specialist. This also highlighted a possible area to which technical assistance from the Regional Offices of the DOH could be directed.

A recent independent review of the IC in the Philippines confirmed that health officials engaged in the process found particular value in the structured analysis of constraints, referring to earlier approaches to defining constraints as haphazard [13]. Some officials observed that their standard approach to understanding system constraints had involved a focus on supply side factors only, thus the bottleneck analysis approach to understanding the root cause of problems by analysing not only supply, but also quality and demand factors, was particularly well received. In one site, the approach to analysis of root causes was reportedly then adopted by the tuberculosis program.

In some cases, the IC analysis challenged existing beliefs about the nature and scope of local constraints and the strategies required. For example, in Eastern Samar, maternal deaths had largely been attributed to “the three delays” [14]. Through the workshop discussion and data review, it became apparent that most maternal deaths took place in hospitals, and resulted from a lack of critical supplies, such as blood, and inadequate staffing. Strategies to overcome these constraints were identified, such as removing the out-of-pocket cost of donating blood, which was considered a key factor in limiting the available supply. The IC process also provided an opportunity to consider new ways of thinking about recognised problems. An example of this was encountered in Pasay City, where greater engagement with the private sector to increase equitable access to maternity services was identified during problem-solving workshops as an alternative to the existing plan to build additional public facilities. However, several stakeholders who were involved in the IC approach and subsequently interviewed two years later reported that the analysis simply confirmed their original understanding and knowledge of constraints, albeit this time with quantifiable estimates [13].

Given the limited number of novel and innovative strategies that actually emerged from the problem-solving or were adopted in practice, it could be argued that the process added little value [13]. However, our experience with officials was that the real value in undertaking the analysis was to help them more systematically ask questions, for example, focusing on the underlying constraints and root causes of demand-side weaknesses in health care as well as the traditional focus on supply-side constraints. Many of the scale-up strategies were based on national guidelines for MNCH. The IC helped officials decide between the range of different scaling-up strategies advocated by the national government, and to tailor different approaches according to local problems, needs and resources. In these cases, local health officials reported that the IC process helped to guide, or at least confirm, their decision-making and the development and prioritisation of different strategies, to validate whether existing assumptions were supported by the data and put numbers to these problems. For example, one of the proposed strategies reflecting recent priorities and approaches supported by the national government is the strengthening of community health teams. The use of evidence and systematic discussion helped determine whether such strategies were indeed the best way to address local health system constraints, and at times the national recommendation was adapted to accommodate the local context. For instance in the two provinces the IC process estimated the funds required to implement the national recommendation to install one midwife per barangay (village) to support community health teams. Given the high observed cost of this strategy and recognising the local human resource shortage, local officials ultimately opted instead to increase midwife numbers to reach a ratio of 1:3 considering this to be a more feasible plan in the short to medium term. The analysis also helped to define the specific local health system inputs and activities that would be required to implement these strategies.

In addition, we noted how the process of devolving responsibility for decision-making can result in simple issues being overlooked. For example, following decentralisation some key medical supplies are no longer being funded by the national government. While the supply of these items had become the responsibility of the local government, the IC discussion and review of data exposed the fact that local officials were still following previous practice of not allocating funding in their local budget. The simple strategy to address this issue was to amend the local budget to include a line item for the relevant supplies and to allocate responsibility for ensuring this additional cost was included in the budget.

Pasay City’s novel strategy of engaging with the private sector to expand access to delivery services had been included in the City’s 2011 AOP, and potentially could have led to substantial savings, but subsequent amendments in the 2012 budget saw a reversion to the earlier strategy of constructing a new public facility. A greater focus on sustaining our partnership with the local government beyond the drafting and submission of the plans would likely have helped retain momentum and interest in the strategies. In retrospect, it is clear that government officials need to expend time, energy and “bureaucratic capital” to convince higher-up decision makers to try new approaches. Simply having the evidence-base of a potentially better approach is not sufficient in itself to achieve step-wise changes in policy. Traditional ways of doing things can be easier and less resource-intensive than using what appears to be an untried mechanism. It is also recognised that, in any setting, there is a range of factors that will impact on prioritisation, plans and funding allocation.

We observed a further example of this in defining strategies around increasing access to family planning. While the evidence for efficacy and cost suggests strategies should focus on expansion of modern methods, in some sites the focus remained on expanding access to less efficacious but more locally acceptable methods. The influence of broader factors such as the political environment in guiding planning and priority-setting has been noted previously [15], and observed in other IC sites [5]. Indeed the 2012 review of the Philippines IC noted that political issues were considered critical in determining what is ultimately included in plans and implemented [13].

## Conclusions

The IC assisted in the thinking about and prioritisation of strategies to respond to identified local problems, and most of those strategies were reflected in the plans and budgets of the participating LGUs. Many of these were approaches being advocated by the national government, thus it is possible that the LGUs may have pursued such strategies through their routine planning mechanisms. The contribution of the IC was to enable these approaches to be adapted to the local setting, provide a higher level of confidence by providing quantitative estimates about projected outcomes and costs, and a more systematic way of analysing the root causes of problems in both the demand and supply side of essential health care. One novel policy option was accepted initially and reflected in initial budget papers, but the option was not pursued, with officials reverting to a more traditional approach.

The lack of data available at the sub-national level is a major impediment to local planning. We undertook highly time- and resource-intensive one-off data collection and collation activities from a broad range of sources in order to identify the best available information for analysis. Such activities are simply not sustainable or replicable for routine planning by LGUs. Supporting longer-term evidence based planning at local level will require significant improvements in the scope and quality of locally-representative health system data, such as health service coverage data. Decentralisation allows greater local flexibility to implement plans that respond to local priorities and needs, but this accordingly requires stronger data systems that allow identification of problems, and monitoring of strategies to improve health service access and health outcomes. The IC process aided in identifying critical data gaps that need to be addressed to further improve sub-national planning and prioritisation for MNCH.

It should be acknowledged that however good the data are, evidence is only one of the factors influencing investments in health. Such evidence-based investments have to fit in with national priorities and at a local level compete with more politically attractive alternatives. In addition, issues relating to decentralisation, such as delays in disbursement of funds from the national level and confused responsibilities for health service delivery and reporting between different levels of government [16], limit the extent to which local level governments can fund and implement their plans. Whilst improving local capacity to produce evidence-based plans and budgets is an important step, parallel efforts at higher levels of government to relieve funding flows and coordination issues are critical for any evidence-based planning approach to have a significant impact on health service delivery.

## Abbreviations

AOP: Annual operational plans; DOH: Department of Health; IC: Investment case; LGU: Local government unit; MNCH: Maternal neonatal and child health; PIPH: Province-wide investment plans for health.

## Competing interests

The authors declare that they have no competing interests.

## Authors’ contributions

All authors contributed to the research for this article. EJS coordinated the multi-country study. AC led the development of the model. SLV, AK and BA led the project team in the Philippines. SLV, BA and EJS wrote the first draft of this article and all authors reviewed subsequent drafts. All authors read and approved the final manuscript.
